# A simple yet reliable assay for chemotaxis in *C. elegans*

**DOI:** 10.17912/micropub.biology.001514

**Published:** 2025-03-11

**Authors:** Samiha Tasnim, Amber Liu, Antony M Jose

**Affiliations:** 1 Cell Biology and Molecular Genetics, University of Maryland, College Park, College Park, Maryland, United States

## Abstract

Animals can move towards or away from an odorant. Here we develop an assay for the nematode *C. elegans* that avoids use of chemical or physical immobilization when measuring response to odorants. We use opposing orientations of rectangular arenas to control for unknown gradients outside the arena and introduce a measure of dispersal to control for locomotion defects and unknown gradients within the arena, enabling the analysis of responses to a variety of chemicals. Using this setup, we found that unfed worms show reproducible movement towards the odorants butanone and benzaldehyde, and away from the odorant nonanone.

**Figure 1. Assay for measuring the response of freely moving C. elegans to odorants:**
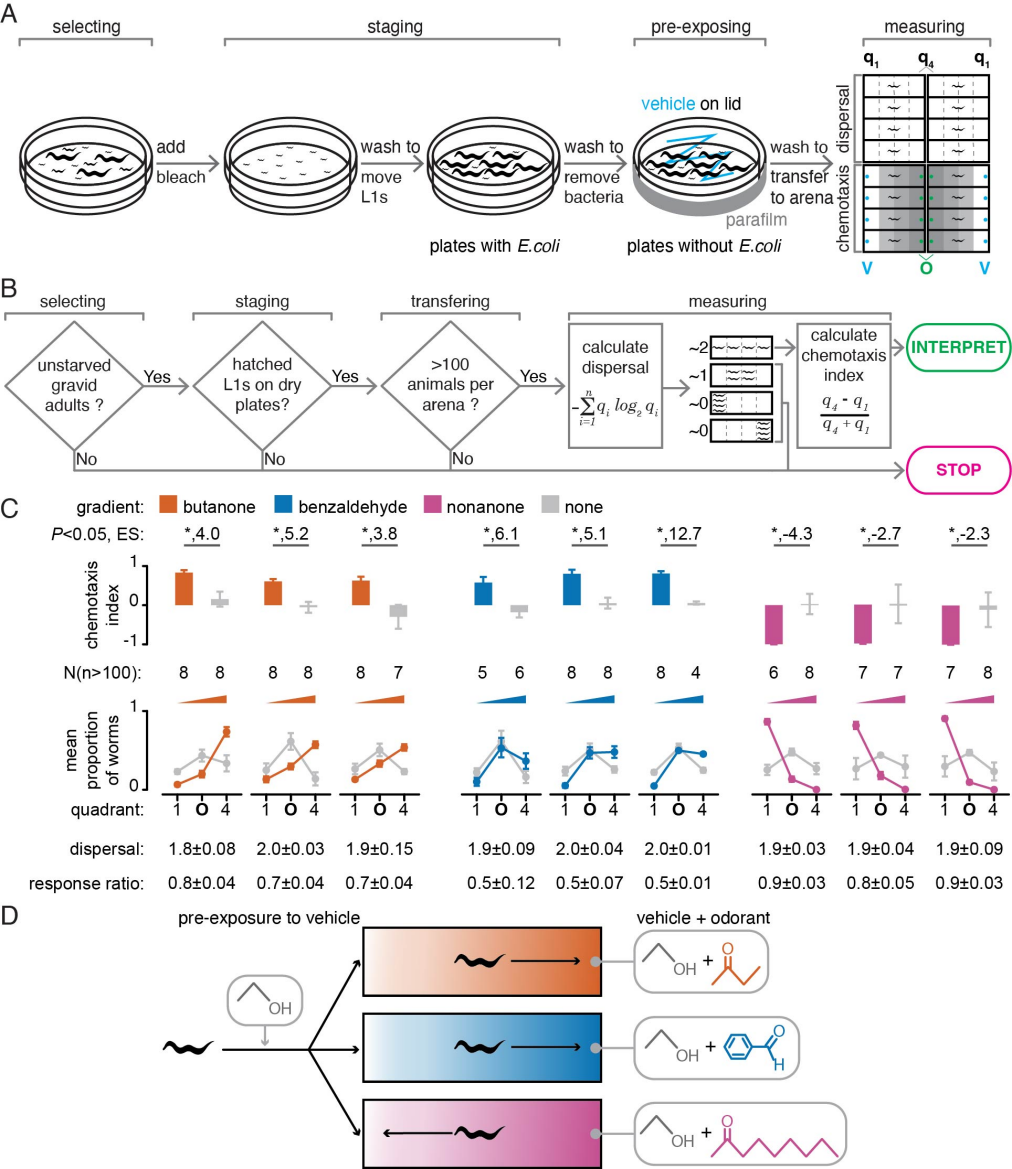
*(A)* Procedure for preparing worms and measuring their response to a volatile odorant. Select plates with unstarved gravid adults for the addition of bleach to dissolve worms while preserving embryos. Move the hatched L1 worms by washing onto plates with *E. coli* OP50 and grow to young adulthood (~96 hrs after bleaching). Wash young adults to remove bacteria and move to plates without *E. coli* to pre-expose them with the vehicle (V; e.g., ethanol) before assaying their response to an odorant in the vehicle (O; e.g., butanone). Transfer pre-exposed worms to the center of each rectangular arena to measure dispersal with no odorants (top) or chemotaxis towards an odorant (q _4_ ) or the vehicle (q _1_ ) (bottom). Count the number of worms in each quadrant (q _1_ to q _4_ ) of the arena using a video taken after 1h. *(B)* A decision chart for interpreting behavior. Results can be interpreted only if sufficient numbers of worms (>100 per plate) of comparable age (young adults) were assayed, and they dispersed uniformly in the absence of odorant (dispersal ~2). In the absence of added odorants, assays where the worms remain in the middle of the arena (dispersal ~1, resulting from attraction to center and/or defective locomotion) or accumulate at one quadrant (dispersal ~0, indicative of response to an unknown gradient in the arena) cannot be interpreted. *(C)* Chemotaxis of wild-type worms is reproducible when tested using gradients of three different odorants. Apparent chemotaxis on arenas without added odorants (grey) paired with chemotaxis measured using three replicate assays for each odorant (butanone, orange; benzaldehyde, blue; nonanone, magenta) are plotted using both calculated chemotaxis index (top) and proportions of animals in extreme quadrants (1 or 4) or near the origin (O; quadrants 2 & 3) (bottom). Effect sizes (ES, Cohen's *d* ), significance (* for *P* <0.05, unpaired t-test), populations tested (N), numbers of worms in each population required for interpretation (n), 95% confidence intervals (error bars), calculated dispersals, and calculated response ratios are shown. Assays remained reproducible when performed on different days (3 different days for butanone and benzaldehyde, and 2 different days for nonanone). *(D)* Summary schematic of the chemotaxis response of *C. elegans* to the tested volatile odorants (attraction to butanone and benzaldehyde, but aversion to nonanone).

## Description

The nematode *C. elegans* is expected to be exposed to a rich variety of odorants when growing on rotting vegetation in the wild (Frézal and Félix, 2015). Responses to individual odorants in the laboratory have been parsed using controlled conditions (Ward, 1973; Bargmann et al., 1993; Hart, 2006) and measurement of neuronal responses in physically constrained animals (Kerr et al., 2000) suggest that single odorants can evoke changes in the activities of multiple neurons (Lin et al., 2023). A normalized difference measure called chemotaxis index ([number near test odorant – number near vehicle control]/[number total]) is widely used in odorant choice assays (reviewed in Queirós et al., 2021), but the odorants and vehicles are often combined with paralytics (e.g., sodium azide (Bargmann et al., 1993)) to immobilize worms or with excess liquid (e.g., ‘buffer pond' (Suzuki et al., 2022)) to collect worms near either choice before counting. Using these conditions, where initial accumulations are captured as choices by trapping the worms, both odorant sensing and associative learning paradigms have been developed. Since trapping worms could overestimate preferences (Albrecht and Bargmann, 2011), it is useful to develop assays that avoid trapping but nevertheless provide a good measure of the response to odorants.

To develop an assay that can measure the behavior of populations of freely moving *C. elegans* , we used rectangular arenas where the ~1-mm worms added to a central origin need to move a minimum of ~20 mm towards or away from an odorant by ~1h to contribute to a chemotaxis index. These criteria ensure that minor preference, chance accumulation, or preliminary exploration is not conflated with a clear response of preference after a period. All worms were prepared for the assay by selecting cohorts, growing them to the same stage (staging), and pre-exposing to vehicle without food (Fig. 1 *A* ). While this pre-exposure to vehicle could influence the chemotaxis response, it serves as a uniform recent experience. The impact of this uniform treatment on the mobility of the worms and the state of arenas before the assay were both evaluated by measuring the ability of the worms to disperse in arenas without any added odorants (Fig. 1 *A* , *top* ). To counter any unknown gradients that may be present in the laboratory, chemotaxis was measured using sets of arenas such that worms in one set must move in the opposite direction to worms in the other set for the same response (Fig. 1 *A* , *bottom* ). In arenas without any salient chemicals, worms are expected to disperse and occupy all sectors uniformly ( *q _1_* to *q _4_* quadrants in Fig. 1 *B* ). Such uniform dispersal results in a calculated entropy (Shannon, 1948) of ~2 (

$$-\sum_{i=1}^{n}q_{i}\log_{2}q_{i}$$,

where *n* = 4 and q _i_ indicates proportions of animals in the i ^th^ quadrant), which can be used as the measure of dispersal. If worms have a locomotion defect or are attracted to the origin, they will accumulate in *q _2_* and *q _3_* , which will reduce the dispersal to ~1. If they are attracted or repulsed by an unknown cue in any one quadrant, the dispersal will be reduced to ~0. Thus, by using identically prepared worms, two sets of rectangular arenas oriented in opposite directions, and a simultaneous measurement of dispersal using a co-cultured population, this assay provides a well-controlled way to ascertain the response to added chemicals without the use of a paralytic while controlling for confounding variables, if any.

Using this assay, we examined odorants that worms have been reported to be attracted to (2-butanone and benzaldehyde) or repulsed by (2-nonanone) (Bargmann et al., 1993). The worms and arenas used in every assay showed a dispersal of ~2 in the absence of added odorants (Fig. 1 *C* , *bottom* ; e.g., Movie S1), which is the prerequisite for interpreting chemotaxis assays (Fig. 1 *B* ; e.g., Movie S2). The responses to all three odorants were in agreement with prior assays and were reproducible when assayed on different days (Fig. 1 *C* , *top* ; 3 different days for butanone and benzaldehyde, 2 different days for nonanone). Specifically, worms were attracted to 10% butanone (median chemotaxis index (CI) of 0.83 and median effect size (Cohen's *d* ) of 4.0) and 20% benzaldehyde (median CI of 0.58 and median effect size of 6.1) but repulsed by 10% nonanone (median CI of -0.99 and median effect size of 2.7).

Interpretation of observed responses was aided by requiring worms to move in opposite directions for the same response, thereby controlling for unknown gradients in the lab, if any, and measuring dispersal in the arena, thereby controlling for locomotion defects and/or unknown gradients within the arena, if any. For example, external gradients along the long axis of each rectangular arena can result in increased variation (e.g., plates with no added odorants paired with assays using nonanone gradients in Fig. 1C, *right* ) because they result in opposing effects in each set of four arenas, ultimately leading to a reduction in overall effect size (e.g., Fig. 1C, nonanone assays). Nevertheless, the response to nonanone appears most robust ( Fig. 1C, *right* ), unlike the response to benzaldehyde ( Fig. 1C, *middle* ), and is therefore potentially useful for genetic screens. While we can infer with confidence when effect sizes are large using this simple assay, the ways to control for confounding variables developed here can also be adapted for more elaborate arenas or workflows that aim to increase throughput (e.g., Fryer et al., 2024).

## Methods

**Worm growth and assays.**

The *C. elegans* wild type Bristol N2 was obtained from the Caenorhabditis Genetics Centre (University of Minnesota, Minneapolis, MN, USA). Worms were grown at 20ºC, and chemotaxis assays were performed at room temperature (~25ºC) as outlined in Figure 1 *A* and scored for both dispersal (Movie S1) and chemotaxis (Movie S2) by capturing movies of the arenas. Detailed methods, including types of plates, concentrations of odorants used, etc., are available online at AntonyJose-Lab/Tasnim_et_al_2024 on GitHub. A replicate of the chemotaxis assay and its paired dispersal were performed together with most replicates being performed on different days and using different cohorts of worms.

**Measures and Inference.**

The proportions of worms in each sector or quadrant (q _i_ with n total) was used to calculate a measure of dispersal that is based on entropy (Shannon, 1948):

dispersal =

$$-\sum_{i=1}^{n}q_{i}\log_{2}q_{i}$$

The proportions of worms in the extreme quadrants (q _1_ and q _4_ ) was noted as the response ratio using the formula

response ratio =

$$\frac{q_{1}+q_{4}}{q_{1}+q_{2}+q_{3}+q_{4}}$$

Chemotaxis towards or away from a volatile odorant was calculated (similar to Hart, 2006) by considering proportions of worms in the quadrant with the odorant in vehicle (q _4_ ) and the quadrant with the vehicle only (q _1_ ) using the formula

chemotaxis index =

$$\frac{q_{4}-q_{1}}{q_{1}+q_{4}}$$

Chemotaxis indices could range from +1 (interpreted as attraction towards the odorant) to -1 (interpreted as aversion to the odorant).

Dispersal, response ratio, and chemotaxis index are all necessary for inference (Table 1). For example, two experiments using 100 worms could result in the same calculated chemotaxis index (= 0.8, say) either when a few worms are in the extreme quadrants (q _4_ = 9, q _3_ = 45, q _2_ = 45, q _1_ = 1; response ratio = 0.1) or many worms are in the extreme quadrants (q _4_ = 45, q _3_ = 35, q _2_ = 15, q _1_ = 5; response ratio = 0.5). The first case (q _4_ = 9, q _1_ = 1) could arise because of a locomotion defect or an attraction to the origin, which could both be revealed by a dispersal <2 and can be used to raise caution in the interpretation of some mutant strains (e.g., *tax-4 osm-9* mutants in Fig. 2 *A* of Fryer et al., 2024). Using worms prepared in the same way for both dispersal and chemotaxis measurements with alternate addition of worms to the respective arenas also controls for variations in worm growth and washing.

Effect sizes were measured using Cohen's *d* (Cohen, 1988), which not only provides a measure of differences in the means (e.g., as indicated in Fryer et al., 2024) but also accounts for the variance. This relative measure is calculated using the formula

effect size =

$$\frac{mean_{test}-mean_{control}}{\sqrt{\frac{sd_{test}^{2}+sd_{control}^{2}}{2}}}$$

Two experiments with the same difference in mean, but different standard deviations will be appropriately reported as having different effect sizes by this measure. For example, for mean _test_ = 0.8 and mean _control_ = 0, the effect size = 8.0 when sd _test_ = sd _control_ = 0.1 but the effect size = 1.6 when sd _test_ = sd _control_ = 0.5. In theory, effect sizes can be influenced by unknown odorants or other cues that are present in the arena before the test odorant is added. For example, the extent of the aversion to nonanone could be modulated by the presence of other attractive cues in the arena even if such unknown cues are uniformly distributed.

**Data, Materials, and Software Availability**

All data generated, the assay protocol used for generating the data, and the code used are available at AntonyJose-Lab/Tasnim_et_al_2024 on GitHub.

**Table 1.** Inference using dispersal, response ratio, and chemotaxis index. The quantitative cut-offs for deciding ‘high' vs. ‘low' for each variable can be set by the researcher based on the effect size and the level of risk tolerance for inference. The scale for each metric is different: response ratio (0 to 1 on a linear scale), chemotaxis (+1 to -1 on a linear scale), and dispersal (0 to 2 on a log scale).

**Table:** 

**Dispersal**	**Response Ratio**	**Chemotaxis Index**	**Inference**
high	high	high	attraction ( *positive chemotaxis index* ) or aversion ( *negative chemotaxis index* ) to the odorant
high	high	low	no locomotion defect ( *high dispersal* ), aversion to the origin on chemotaxis plates only and/or attraction to the vehicle ( *low chemotaxis index and high response ratio* )
high	low	high	no locomotion defect ( *high dispersal* ), attraction to the origin and/or aversion to vehicle on chemotaxis plates only ( *low response ratio* ) overcome by attraction ( *positive chemotaxis index* ) or aversion ( *negative chemotaxis index* ) to the odorant
low	high	high	locomotion defect and/or attraction to the origin on dispersal plates only overcome ( *high response ratio* ) by attraction ( *positive chemotaxis index* ) or aversion ( *negative chemotaxis index* ) to the odorant
high	low	low	no locomotion defect ( *high dispersal* ), attraction to the origin on chemotaxis plates only and/or aversion to the vehicle ( *low chemotaxis index and low response ratio* )
low	low	high	locomotion defect and/or attraction to the origin across all plates with attraction ( *positive chemotaxis index* ) or aversion ( *negative chemotaxis index* ) to the odorant
low	high	low	locomotion defect and/or attraction to the origin on dispersal plates overcome by aversion to origin and/or attraction to the vehicle ( *low chemotaxis index but high response ratio* )
low	low	low	locomotion defect and/or attraction to the origin across all plates

## Data Availability

Description: Protocol, Data, and Code for plotting. Resource Type: Software. DOI: https://doi.org/10.22002/b92ex-pfe21 Description: Movie S1. Representative video panning through 2 sets of 4 arenas with a marked lid underneath the arena at the end of a dispersal assay. Resource Type: Audiovisual. DOI: https://doi.org/10.22002/gcef8-rnd46 Description: Movie S2. Representative video panning through 2 sets of 4 arenas with a marked lid underneath the arena at the end of an assay for the chemotaxis response to nonanone. Resource Type: Audiovisual. DOI: https://doi.org/10.22002/swhm7-eaa57
